# The “slower” the better

**DOI:** 10.1007/s40618-014-0065-x

**Published:** 2014-03-22

**Authors:** L. Pala, C. M. Rotella

**Affiliations:** 1 Endocrine Unit, Careggi University Hospital, Florence, Italy; 2 Department of Biomedical Experimental and Clinical Sciences, University of Florence, Obesity Agency, Careggi University Hospital, Viale Pieraccini 6, 50134 Florence, Italy

A new formulation of metformin: metformin extended-release (ER) is now available, with different formulations in each country and it appears relevant to discuss the management of this drug in clinical practice. Metformin, an oral biguanide hypoglycemic agent, is an efficacious tool in the treatment of type 2 diabetes mellitus. Metformin’s efficacy, security profile, benefic cardiovascular and metabolic effects make this drug as the first agent of choice in the treatment of type 2 diabetes, together with lifestyle modifications [1]. Type 2 diabetes is characterized by impaired insulin secretion and insulin resistance. Insulin resistance in the fasting state induces an increase in hepatic gluconeogenesis and induces hyperglycemia in the early morning. Metformin, as its major effect, decreases hepatic glucose output lowering fasting glycaemia and, secondarily, it increases glucose uptake in peripheral tissues. It is generally well tolerated, despite the fact that the most common adverse effects are gastrointestinal ones, which may be tampered by dose titration. In monotherapy metformin decreases HbA1c levels by 0.6–1.0 % and this is not accompanied by hypoglycemia in the large majority of patients. Metformin is neutral with respect to weight or, possibly, induces a modest weight loss. The UKPDS has demonstrated a beneficial effect of metformin therapy on CVD outcomes [2]. Severe renal dysfunction is considered a contraindication to metformin use, because it may increase the risk of lactic acidosis; therefore, therapy should be discontinued if the estimated glomerular filtration rate falls below 30 ml/min. The pharmacokinetic characteristics of the conventional immediate-release (IR) formulation of metformin need three times daily dosing. Metformin is primarily used to treat type 2 diabetes, but it is also beneficial in the treatment of other metabolic diseases such as the polycystic ovarian syndrome (PCOS) and the non alcoholic fat liver disease (NAFLD).

Metformin ER was obtained using a Gelshield diffusion system that gives to metformin a slower absorption than IR with a maximum plasma concentration of 7 h versus 3. Metformin ER releases the active drug through hydrated polymers which expand safe uptake of fluid, prolonging gastric transit and slowering drug absorption in the upper gastrointestinal tract [3]. The extent of absorption is equivalent for both formulations. The maximum plasma concentrations (Cmax) of metformin ER 2,000 mg once daily is higher than metformin 1,000 mg twice a day. Metformin ER is well tolerated at single dose and there is no accumulation with multiple dose administration [4].

Many studies have demonstrated that once or twice daily administration of ER metformin is as safe and efficacious as twice daily IR metformin, overall providing continued glycemic control for up to 24 weeks of treatment [5]. A prospective open label study has shown that metformin ER was efficacious on blood glucose, lipid profile and blood pressure [6]. While metformin IR maintains efficacious concentration up to 6 h, the efficacy of metformin ER is about 12 h. This means that if in the current clinical setting IR metformin needs three time daily dosing, we should use twice daily ER metformin dose because in the current clinical setting, twice daily ER metformin is equivalent to a three time daily dose of IR metformin and once a day ER metformin is equivalent to twice daily IR metformin. In Table 1 a comparison between the characteristics of metformin ER versus those of metformin IR are reported.

**Table 1:** Comparison between metformin ER and metformin IR

	Metformin ER	Metformin IR
Administration	Oral	Oral
Duration (hours)	12	6
Daily times dosing	2	3
Maximum plasma concentration (hours)	7	3
GI side effects	−	+
HbA1c Reduction (%)	0.6–1	0.6–1

## Which advantages presents metformin ER with respect to metformin IR?

First of all, it enables a less number of doses daily and this improves the patient compliance to therapy. Besides, metformin ER has a slow absorption without a rapid peak in blood stream and this produces less side gastrointestinal effects [7–9]. The last, but not the least, advantage is to reduce glycemia in the early morning which ameliorates the glycemic control of patients.

## Conclusions

Metformin ER is associated by less gastrointestinal side effects. Moreover, it needs less frequent daily doses because of the drug pharmacokinetics, but, despite the opinion of some authors [4–6] suggesting the use metformin ER once a day, in our opinion it is strongly recommendable to use at least two time daily dose because of all the reasons described above. These considerations, all together, contribute to improve the patient compliance to the therapy. In addition, since there are different formulations of metformin ER in each country, we can suggest to replace the total daily dose of metformin IR with the same daily dose of metformin ER divided twice a day. We do not recommend the use of metformin ER in once daily dosing because of its pharmacokinetics, in fact, if this drug has a duration of 12 h, under these condition it is not able to cover neither the inter prandial phase in a day, nor the morning hyperglycemia. Moreover in PCOS and NAFLD, diseases in which it is important to reduce insulin resistance during a 24 h period, the use of metformin ER could improve the efficacy of this therapeutical approach. Metformin ER could have also beneficial effects in prediabetes conditions and in obesity [10].
